# RNAscope in situ hybridization-based method for detecting *DUX4* RNA expression in vitro

**DOI:** 10.1261/rna.070177.118

**Published:** 2019-09

**Authors:** Gholamhossein Amini Chermahini, Afrooz Rashnonejad, Scott Q. Harper

**Affiliations:** 1 Center for Gene Therapy, The Research Institute at Nationwide Children's Hospital, Columbus, Ohio 43205, USA; 2 Department of Pediatrics, The Ohio State University, Columbus, Ohio 43205, USA

**Keywords:** FSHD, DUX4, RNA in situ hybridization, RNA, RNAscope

## Abstract

Facioscapulohumeral muscular dystrophy (FSHD) is among the most common forms of muscular dystrophy. FSHD is caused by aberrant expression of the toxic *DUX4* gene in muscle. Detecting endogenous *DUX4* in patient tissue using conventional methods can be challenging, due to the low level of *DUX4* expression. Therefore, developing simple and trustworthy *DUX4* detection methods is an important need in the FSHD field. Here, we describe such a method, which uses the RNAscope assay, an RNA in situ hybridization (ISH) technology. We show that a custom-designed RNAscope assay can detect overexpressed *DUX4* mRNA in transfected HEK293 cells and endogenous *DUX4* mRNA in FSHD patient-derived myotubes. The RNAscope assay was highly sensitive for tracking reductions in *DUX4* mRNA following treatment with our therapeutic mi405 microRNA, suggesting that RNAscope-based *DUX4* expression assays could be developed as a prospective outcome measure in therapy trials. This study could set the stage for optimizing and developing a new, rapid RNA ISH-based molecular diagnostic assay for future clinical use in the FSHD field.

## INTRODUCTION

Facioscapulohumeral muscular dystrophy (FSHD) is among the most common types of muscular dystrophy with a prevalence of about 5–12 per 100,000 individuals (Deenen et al. 2014). FSHD shows autosomal dominant (FSHD1) or digenic (FSHD2) inheritance and the disease often manifests with slowly progressive muscle weakness involving the facial, scapular, upper arm, lower leg, and hip girdle muscles (Statland and Tawil 2014, 2016). Asymmetrical involvement can occur. Although the disease was named for the muscle groups most commonly affected, there is no universal pattern of muscle involvement and patients can show wide variability in symptoms. Clinical features often become evident in the second decade of life although age-at-onset and rate of progression may also vary (Statland and Tawil 2014, 2016).

FSHD is caused by aberrant expression of the *double homeobox protein 4* (*DUX4*) gene (Lemmers et al. 2010, 2012). The *DUX4* gene is located within repetitive elements, called D4Z4 repeats, located on the subtelomere of chromosome 4q35. Except in early embryogenesis or adult testis, this region is normally embedded in heterochromatin, and therefore *DUX4* is not transcribed. In individuals with FSHD, the 4q35 region is epigenetically derepressed (Lemmers et al. 2010, 2012). Several genetic conditions can trigger a change in the epigenetic structure of the 4q35 D4Z4 region from a heterochromatin state to a more open, euchromatin-like state. These derepressing epigenetic lesions permit *DUX4* transcription, and if this occurs on a chromosome 4 allele containing an adjacent poly A signal for *DUX4* (called the 4qA haplotype), the mRNA is polyadenylated and can be translated by the ribosome. The resultant DUX4 protein is toxic to muscle, cultured myocytes, and other nonmuscle cells in vitro (Lemmers et al. 2010, 2012; Wallace et al. 2011; Giesige et al. 2018).

Despite causing potentially devastating effects in muscles of FSHD patients, and increased cell death when expressed in vitro, *DUX4* expression is relatively rare (Kowaljow et al. 2007; Snider et al. 2009, 2010; Wallace et al. 2011; Ferreboeuf et al. 2014). Indeed, *DUX4* may typically be present in only a small percentage (0.1%–0.01%) of myonuclei from FSHD patient cell lines (Snider et al. 2010), and this relative scarcity has posed challenges for detecting *DUX4* messenger RNA or DUX4 protein reliably using methods like PCR, western blotting or immunohistochemistry in vivo. For example, some PCR assays have required very large amounts of starting material and/or 55–70 PCR cycles to detect reverse-transcribed *DUX4* expression from FSHD patient materials (Dixit et al. 2007; Jones et al. 2012). At the protein level, the currently available DUX4-specific antibodies have so far not been useful for reliably detecting endogenous DUX4 protein by western blot or immunofluorescence staining in patient biopsy material. In addition to impacting basic research, the difficulty to reliably detect *DUX4* expression could pose challenging for future prospective clinical trials involving *DUX4* inhibition therapies, where *DUX4* levels would be useful as a therapeutic outcome measure.

As an alternative approach to detect *DUX4* expression, here we tested a custom in situ hybridization (ISH) method using a powerful and highly specific technology called RNAscope (Wang et al. 2012). RNAscope relies upon “double Z” probe technology and specific signal amplification steps that virtually eliminate the background noise often encountered with traditional ISH approaches (Wang et al. 2012; Deleage et al. 2018). As a result, the RNAscope signal amplification method permits sensitive detection of low abundance RNAs, while also allowing localization of mRNAs in individual cells (Wang et al. 2012; Deleage et al. 2018). Because *DUX4* has low abundance and sporadic expression, we reasoned that RNAscope could be an ideal method for *DUX4* detection. At the time we began this study, the vendor (ACDBio) had not developed *DUX4*-targeted RNAscope probes and we therefore initiated custom designed *DUX4* RNAscope probes to detect overexpressed and endogenous *DUX4* mRNAs in vitro. These probes are now available as catalog items at ACDBio. The entire protocol from cell fixation to imaging takes about 8 h and can be accomplished during 1 d. Our results demonstrate proof-of-principle for using RNAscope ISH technology to detect *DUX4* mRNA at overexpressed and endogenous levels of expression in human cells, and set the stage for translating the method for detecting *DUX4* in human FSHD biopsies.

## RESULTS

### Detection of overexpressed *DUX4* in transfected human HEK293 cells

**FIGURE 1.**
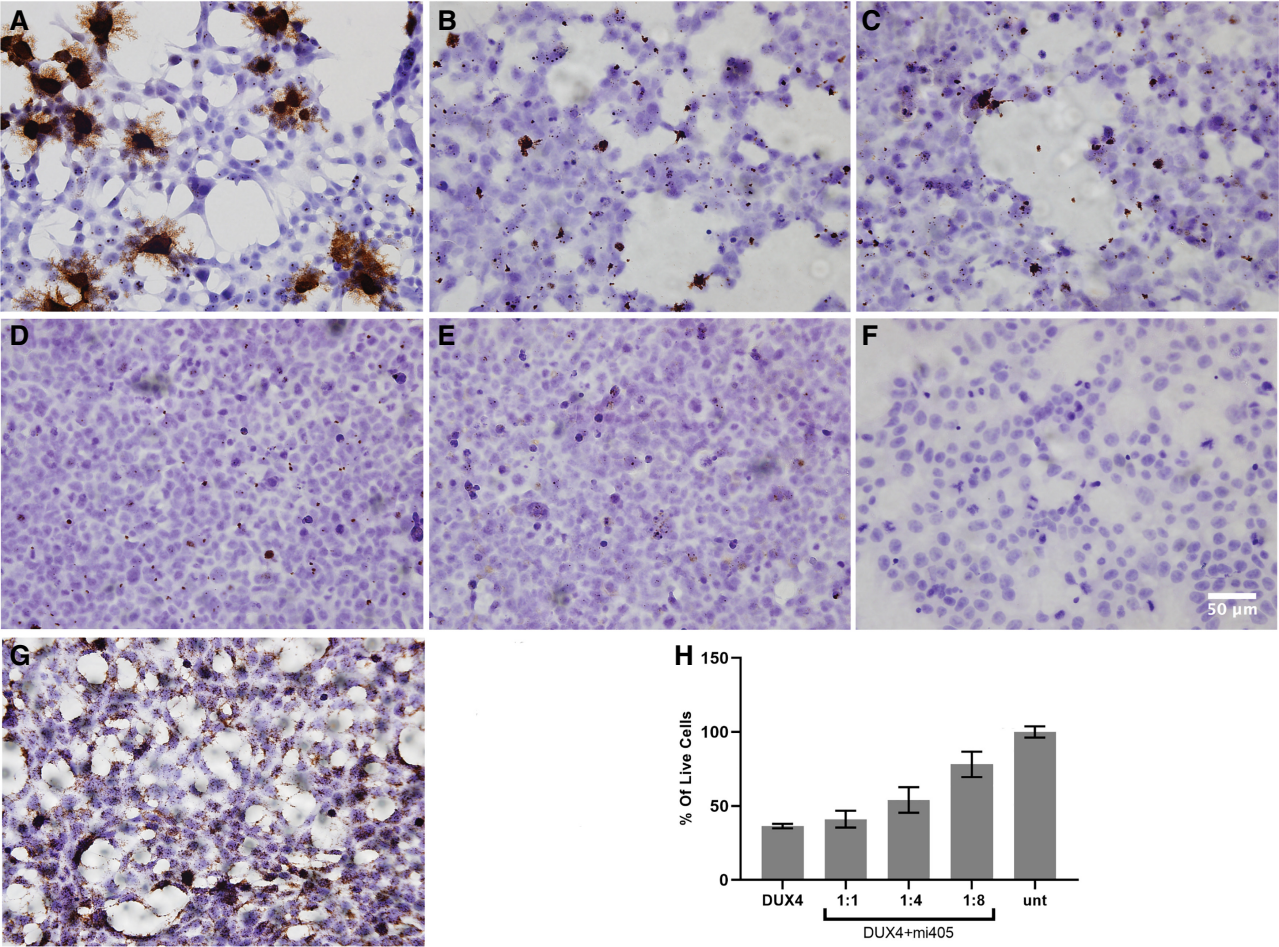
RNAscope specifically detected overexpressed *DUX4* mRNA in transfected HEK293 cells. (*A*) *DUX4*-transfected cells showed punctate brown staining with RNAscope assay, while controls did not. Decreased brown staining with increased doses of *DUX4*-targeted microRNAs (mi405) demonstrated specificity. (*B*–*D*) *DUX4* signal after cotransfection of HEK293 cells with CMV.DUX4 and U6.mi405 plasmids at 1:2, 1:4, and 1:8 ratios. (*E*) Absence of brown *DUX4* signal in untransfected HEK293 cell line. (*F*) RNAscope negative control stain. (*G*) Housekeeping gene *PPIB* was detected in all HEK293 cells and served as a positive control for the assay. (*H*) Cell viability assay demonstrated that mi405 reduced the levels of overexpressed *DUX4* mRNA, and significantly protected cells from death at 1:4 and 1:8 weight ratios (*N* = 3 independent experiments performed in triplicate); *P* < 0.0001, ANOVA. 40× objective. Scale bar, 50 microns.

Our goal was to develop a novel and efficient technique for detecting *DUX4* mRNA in mammalian cells. To do this, we tested the ability of custom-made *DUX4*-targeted RNAscope probes to detect overexpressed *DUX4* mRNA in HEK293 cells transfected with a CMV.DUX4 expression plasmid. Sixteen hours later, we then fixed cells and stained with the RNAscope assay, using a diaminobenzidine (DAB) reagent which stains hybridized target mRNAs brown. We found that *DUX4*-transfected cells showed abundant, punctate brown dots and also some spider-like projections that were evident throughout the slides (Fig. 1). We detected no *DUX4* signal in untransfected HEK293s (Fig. 1). As a positive control for the RNAscope assay, we used a probe targeting the housekeeping gene *PPIB* (*peptidylprolyl isomerase B*), which was provided with the RNAscope assay kit. Importantly, to demonstrate the specificity of our custom *DUX4* RNAscope probes to detect *DUX4* mRNA, we cotransfected HEK293 cells with the CMV.DUX4 expression plasmid and an U6 promoter-driven microRNA, called mi405, which we have previously shown significantly knocks down *DUX4* mRNA using an RNA interference (RNAi) mechanism (Wallace et al. 2012, 2018). *DUX4*-expressing cells transfected with U6.mi405 showed microRNA-dose dependent reduction in the amount and intensity of brown punctate staining, thereby supporting the specificity of the *DUX4* probe (Fig. 1). In addition, consistent with our prior work, qualitatively we observed that increasing amounts of the *DUX4*-targeted microRNA protected HEK293 cells from DUX4-dependent cell death, in a dose-dependent fashion (Fig. 1). Upon quantification with trypan blue cell viability assay, we found that mi405 coexpression produced dose-dependent protection from DUX4-induced cell death (Fig. 1).

### Detection of endogenous *DUX4* in myotubes derived from FSHD patient biopsies

**FIGURE 2.**
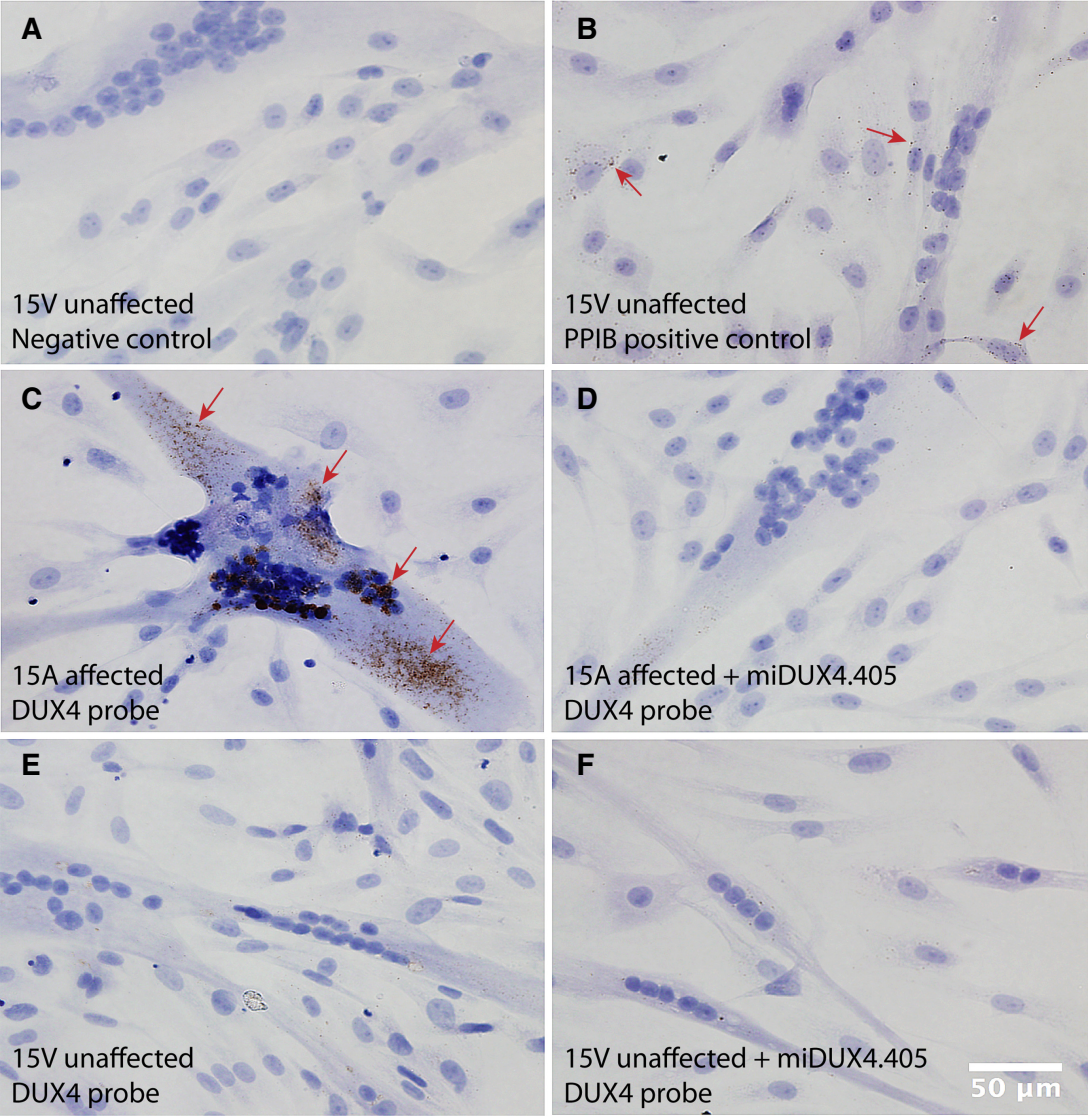
RNAscope detects endogenous *DUX4* mRNA in 15A FSHD myotubes. FSHD 15A myotubes demonstrated higher amounts of *DUX4* mRNA compared to non-FSHD 15V myotubes, as determined by RNAscope staining. Arrows indicate brown punctate signal. (*A*) Negative control stain. (*B*) 15V cell line stained with the housekeeping gene *PPIB* served as a positive control for the assay. (*C*) *DUX4* expression in FSHD 15A myotubes was reduced or absent in (*D*) 15A cells transfected with U6.mi405 microRNAs. (*E*) Very weak or absent signal was present in the unaffected 15V cell line alone and in (*F*) 15V transfected with U6.mi405 plasmid.

**FIGURE 3.**
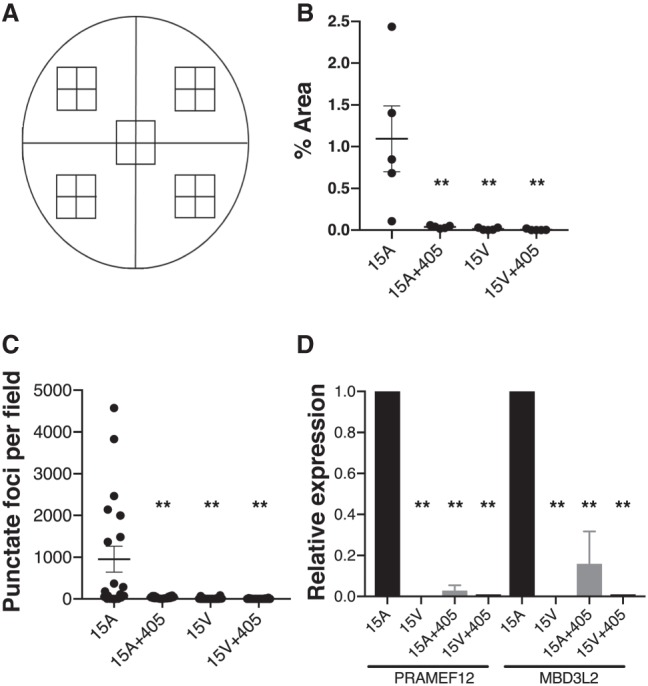
Quantification of *DUX4* RNAscope signal and DUX4-activated biomarkers. (*A*) Schematic representation of a cover slip on which FSHD and control myoblasts were grown and differentiated to myotubes. For each sample, a total of 20 image fields were taken from five zones (four quadrants and one central zone). *DUX4* RNAscope signal was quantified as demonstrated in the video in Supplemental Figure 2. (*B*) Percent area of *DUX4* signal in one representative experiment (from three independent experiments). Each data point represents total % area per zone. In this experiment, *DUX4* signal was significantly elevated in 15A myotubes compared to unaffected 15V controls and to 15A cells transfected with the mi405 therapeutic microRNA. (**) Represents significant differences from untreated 15A FSHD cells (*P* < 0.01; ANOVA). (*C*) Data presented here indicate that despite low abundance relative to the entire 15A culture, the ∼1% of cells showing *DUX4* signal often expressed high amounts of *DUX4* mRNA. Here, each data point represents the number of *DUX4* positive foci per field in a representative culture. One *DUX4* focus was defined as 16 pixels, which was the minimum visible *DUX4* signal. Again, *DUX4* signal was absent or very low in unaffected 15V cells, as well as affected 15A cells transfected with mi405 plasmid. (**) Represents significant differences from untreated 15A myotubes (*P* < 0.01; ANOVA). (*D*) QPCR assays of DUX4-activated biomarkers, *PRAMEF12* and *MBD3L2*, in 15A and 15V cells. Biomarker expression was used to confirm the specificity of the RNAscope assay and knockdown of endogenous *DUX4* by miDUX4.405. Both biomarkers were significantly elevated in affected 15A myotubes, but absent or virtually absent in 15V cells (no signal at cycle 40). *PRAMEF12* and *MBD3L2* were significantly reduced in 15A cells transfected with miDUX4.405 plasmid. Data were acquired from *N* = 3 independent experiments, with each QPCR assay performed in triplicate. (**) Represents significant differences from untreated 15A myotubes (*P* < 0.01; ANOVA).

We next determined the sensitivity of the RNAscope assay to detect endogenous *DUX4* in FSHD patient cells. To do this, we differentiated the FSHD-affected 15A cell line and an unaffected paired control (15V) for 4 d, and then performed RNAscope assay on fixed myotubes (Jones et al. 2012). We found abundant punctate brown staining in the 15A cells, and absent or very faint background staining in unaffected 15V myotubes (Fig. 2). We confirmed this staining pattern in four other cell lines derived from FSHD patient biopsies or unaffected family members (affected 16A and 17A; unaffected 16U and 17U) (Supplemental Fig. 1). Specifically, like the 15A–15U pairs, *DUX4* RNAscope signal was detected in FSHD-affected cells (16A and 17A) but absent or at background levels in unaffected cells (16U and 17U). *PPIB* stain was again used as a positive control for the assay. To determine specificity of the RNAscope assay to detect endogenous *DUX4*, we electroporated cells with the U6.mi405 plasmid prior to differentiation, and then performed RNAscope staining. We found that mi405 treatment reduced RNAscope signals in differentiated myoblast cells, suggesting that the method was both sensitive and specific for detecting endogenous *DUX4* mRNA (Figs. 2, 3).

We next quantified the *DUX4* RNAscope signals in 15A and 15U cells using a microscopic morphometry method. Specifically, we developed a grid system and took digital photomicrographs of 20 microscopic fields within five zones for every cell culture sample (Fig. 3A). We then measured *DUX4* signal using Fiji/ImageJ software quantification (Wayne Rasband, National Institutes of Health) essentially following manufacturer's instructions (ACDBio), and generated a video example of the quantification method (Supplemental Fig. 2). We found that *DUX4* signal was significantly higher in 15A cells compared to 15V, where it was virtually absent, which was consistent with a previous study using other methods of detection (Jones et al. 2012). Despite increased *DUX4* signal in 15A compared to unaffected 15V cells, only about 1% of the total area of 15A myotubes showed *DUX4* RNAscope signal (Fig. 3B). This sporadic expression in only a small percentage of cells was also consistent with previous work (Snider et al. 2010). Nevertheless, in those rare cells where *DUX4* was expressed, signals were often very high (Fig. 3C). To illustrate this, we quantified the number of *DUX4*-positive punctate foci, where one focus was defined as 16 pixels, as this was the smallest discernible RNAscope signal above background. Using this method, we found that some fields showed almost no signal, while others had thousands of DUX4+ foci per field (Fig. 3C). To confirm the reproducibility of this quantification method, a second person independently counted the same microscopic fields using the same methodology but with a blinded approach. Specifically, the second operator was provided individual images randomly and blinded across all groups. Importantly, the results were concordant between the first and second quantification (Fig. 3B,C; Supplemental Fig. 3). As a confirmation of signal specificity, *DUX4* signal was significantly reduced to background in mi405-transfected 15A cells (Figs. 2, 3B,C). These data were consistent with our previous data showing that the *DUX4*-targeted mi405 construct significantly knocked down overexpressed *DUX4* mRNA in human cells and in a DUX4 mouse model (Wallace et al. 2012, 2018). As a final confirmation of *DUX4* expression in affected and unaffected cell lines as well as the impacts of *DUX4* knockdown by miDUX4.405, we used quantitative RT-PCR to measure expression of the DUX4-activated human biomarkers *PRAMEF12* and *MBD3L2* in cultured myotubes. We found that both biomarkers were present in affected cells (15A, 16A, 17A) but absent or extremely low in unaffected cells (15V, 16U, 17U), and that affected cells treated with mi405 showed significantly reduced or absent DUX4 biomarker expression (Fig. 3D; Supplemental Fig. 4).

## DISCUSSION

The FSHD field has made tremendous progress during the last decade, including the publication of a unified model of pathogenesis, development of numerous animal and cell models, and the emergence of several new promising therapeutic strategies. As a sign that the FSHD field has turned a corner toward translational work, there are now several efforts to establish clinical outcome measures for emerging therapeutic studies. Unfortunately, to our knowledge, at this time there are not established methods for reliably detecting endogenous *DUX4* expression in clinical samples. As a result, it is currently infeasible to use *DUX4* expression as a clinical biomarker for FSHD, although there has been considerable effort to identify and detect DUX4-associated biomarkers for use as an indirect indication of *DUX4* gene expression (Yao et al. 2014; Rickard et al. 2015; Eidahl et al. 2016; Jagannathan et al. 2016; Heuvel et al. 2018). Although these will likely prove very useful, we propose that having the ability to directly measure *DUX4* expression would be optimal for FSHD therapeutic strategies that are aimed at reducing *DUX4* levels, such as an RNAi-based gene therapy we are working to translate (Wallace et al. 2012, 2018). Several approaches for *DUX4* detection have been used in recent years. As mentioned, this is difficult due to the scarcity of *DUX4* expression, even in FSHD patient cells and tissues. Although no published work has shown DUX4 protein detection in FSHD biopsies, it can be found in rare nuclei (generally <1%) of differentiated myocytes isolated from FSHD patients using indirect immunofluorescence staining with DUX4 antibodies. This scarcity and nonuniformity in expression within a single culture plate extends to *DUX4* mRNA, where again *DUX4* is absent in most cells (Heuvel et al. 2018). This phenomenon makes a global transcript detection method like QPCR challenging, as it would not be able to indicate how many cells on the plate were expressing *DUX4*, and there would be a dilution effect on *DUX4* signal in a total RNA population since most cells in the culture would not express it. It may therefore be preferable to have the ability to detect *DUX4* mRNA and—importantly for therapy studies—accurately quantify *DUX4* levels in single cells. This could be done using complex cutting edge methods like single-cell RNA sequencing (Heuvel et al. 2018), or ISH, which is what we developed here.

We note that *DUX4* was detected by traditional immunofluorescence-based ISH in one other study, but that single paper was not focused on assay development, and the usefulness of ISH for broad application and quantification was not determined (Ferreboeuf et al. 2014). Specifically, *DUX4* signal was shown only for three nuclei at high power, and no quantification or validation of specificity were pursued. In contrast, we extensively characterized the specificity of a new *DUX4*-targeted RNAscope method here, and described in-depth a strategy for quantification using light microscopy and publicly available ImageJ software. Specifically, as a first step to develop a new assay for *DUX4* detection, we tested custom-made (but now available) RNAscope probes to detect overexpressed *DUX4* in transfected HEK293s, and importantly, endogenous *DUX4* transcripts in FSHD patient-derived myoblasts. Our results in HEK293 cells, which do not naturally express *DUX4*, demonstrated that the assay was able to detect *DUX4* with high specificity. In particular, we observed *DUX4* mRNA signal only in cells transfected with CMV.DUX4 plasmid transfection, and did not detect signal in untransfected cells. Importantly, as another indication of specificity, we also found that *DUX4* signal decreased with increasing doses of our therapeutic miDUX4.405 construct, and that this reduced *DUX4* signal correlated with increased protection from cell death, compared to *DUX4*-only controls. We also showed sensitivity of the assay to detect comparatively low levels of endogenous *DUX4* transcript in patient-derived myotubes, and again confirmed probe specificity in these cells as demonstrated by reduced *DUX4* signal, as well as DUX4-activated biomarkers (*MBD3L2* and *PRAMEF12*) in FSHD patient cells transfected with U6.miDUX4.405 plasmids. Finally, we used a blinded approach to support that the quantification method was reproducible among two different operators. Our future work with this assay will focus on detecting *DUX4* in vivo, using muscle sections from an FSHD mouse model and biopsies from human patients.

In conclusion, we found that RNAscope is a highly sensitive method for detecting *DUX4* mRNA in vitro, and may enable us to develop a new, rapid RNA ISH-based molecular diagnostic assay for FSHD. Importantly, the *DUX4* RNAscope assay was able to detect reductions in *DUX4* mRNA following treatment with our therapeutic microRNA, miDUX4.405. These results suggested that RNAscope may be developed for use as a clinical outcome measure after *DUX4*-modulating treatments.

## MATERIALS AND METHODS

### Transfection of DUX4 and miDUX4.405 expression plasmids into human HEK293 cells

HEK293 cells were seeded in triplicate on coverslips in a 24-well plate at a density of 200,000 cells per well. Cells were incubated in Dulbecco's modified eagle medium (DMEM) (Lonza) supplemented with 10% fetal bovine serum (Hyclone), l-glutamine (Invitrogen) and penicillin/streptomycin (Sigma-Aldrich) at 37°C in 5% CO_2_. Upon reaching 70% confluency, cells were transfected with 500 ng of CMV.DUX4 expression plasmid using Lipofectamine-2000 (Thermo Fisher Scientific), according to manufacturer's instructions. Sixteen hours after transfection, cells were fixed with 4% PFA and RNAscope staining was performed following manufacturer's instructions (detailed below). To demonstrate specificity, we performed a *DUX4* dose-response in which *DUX4* was knocked down with our lead therapeutic *DUX4*-targeted microRNA, called mi405. For those experiments, cells were cotransfected with CMV.DUX4 and U6.mi405 expression plasmids at 1:1, 1:4, and 1:8 weight ratios (Wallace et al. 2012, 2018).

### Culturing of FSHD patient myotubes

We used previously described FSHD patient myotubes to test the ability of our custom RNAscope assay to detect endogenous *DUX4* (Jones et al. 2012). Human immortalized myoblasts 15V, primary 16U and 17U (unaffected) and immortalized 15A, primary 16A and 17A (FSHD affected) were seeded with 4:1 ratio of DMEM: Medium 199 supplemented with 15% fetal bovine serum (Hyclone), 30 ng/mL zinc sulfate (Fisher Scientific), 1.4 µg/mL Vitamin B12 (Sigma-Aldrich), 55 ng/mL dexamethasone (Sigma-Aldrich), 2.5 ng/mL human growth factor (Chemicon International), 10 ng/mL fibroblast growth factor (BioPioneer HRP) and 20 mM HEPES (Sigma-Aldrich). For elucidating specificity of the *DUX4* RNAscope probe, 15V and 15A myoblasts were transfected with U6.mi405 plasmid via electroporation (Lonza) and then 24 h later, switched to differentiation media (4:1 ratio of DMEM:Medium 199 supplemented with 15% KOSR, 2 mM l-glutamine, 1% antibiotics/antimycobiotics [cat. no. 15-240-096 Gibco], 1 mM sodium pyruvate, 20 mM HEPES buffer) for up to 7 d.

### RNAscope staining

Cells were grown on cover slips, which were then mounted on microscopy slides for RNAscope assay. Cells were washed twice with PBS, then fixed with 4% PFA (Fisher Scientific) for 30 min at room temperature, and finally dehydrated with 50%, 70%, and 100% ethyl alcohol gradients for 5 min each at room temperature. Cells were then rehydrated with 70% and 50% ethyl alcohol gradients for 2 min each and finally treated with PBS for 10 min, followed by hydrogen peroxide (cat. no. 322335 ACDBio) and protease III (cat. no. 322337 ACDBio) at room temperature for 10 min each, and then washed with PBS. Probes were then added for 2 h at 40°C within a humidity control chamber. Prior to initiating this study, a DUX4 probe did not exist, and we worked with ACDBio to have one custom developed. It is now listed in the ACDBio catalog as Hs-DUX4-No-XMm, NM_001306068.2, target region: 36–1689 (cat. no. 498541). Signal amplification and detection reagents (cat. no. 322310 ACDBio) were applied sequentially and incubated in AMP 1, AMP 2, AMP 3, AMP 4, AMP 5, and AMP 6 reagents, for 30, 15, 30, 15, 30, 15 min, respectively. Before adding each AMP reagent, samples were washed twice with washing buffer (cat. no. 310091 ACDBio). The samples were then counterstained with 50% Gill's hematoxylin I (cat. no. HXGHE1LT, American Master Tech Scientific) for 2 min at room temperature, rinsed with tap water, placed in 0.02% ammonia water, followed by another tap water rinse. Samples were then dehydrated with 70% and 100% ethyl alcohol gradients for 2 min each, followed by xylene treatment for 5 min (cat. no. X3P-1GAL, Fisher Scientific). Mounting media and cover slips were then added to slides for imaging. Images were captured using an Olympus DP71 microscope.

### RNAscope quantification

For quantification, 20 images per sample were taken from a total of five zones (four quadrants and a central zone). Four 100× images were taken from a random area within each zone. DUX4 RNAscope signals were quantified using ImageJ (Fiji) software as directed by the vendor's guidelines (TS 46-003/Rev A/Date 6212018). Detailed quantification procedures are shown in the Supplemental Video. The amount of the detected signal compared to the total area of each image was calculated using Image J and plotted as both percent area and pixel units, where 16 pixels was considered equivalent to one *DUX4* mRNA focus.

## SUPPLEMENTAL MATERIAL

Supplemental material is available for this article.

## Supplementary Material

Supplemental Material
