# Supplementary material for: RNAscope in situ hybridization-based method for detecting DUX4 RNA expression in vitro
Source: RNA. 2019 Sep;25(9):1211–7. doi: 10.1261/rna.070177.118 (PMC6800509; doi:10.1261/rna.070177.118)
Supplement: Supplemental Material [file supp_25_9_1211__index.html]

RNAscope in situ hybridization-based method for detecting DUX4 RNA expression in vitro — RNAscope in situ hybridization-based method for detecting DUX4 RNA expression in vitro — Supplemental Material 

# RNAscope in situ hybridization-based method for detecting *DUX4* RNA expression in vitro

## Supplemental Material

- Supplemental\_Figure\_1.tif
- Supplemental\_Figure\_2\_mov.mp4
- Supplemental\_Figure\_3.tif
- Supplemental\_Figure\_4.tif
- Supplemental\_Legends.docx
