# Supplementary figures and images for: RNAscope in situ hybridization-based method for detecting DUX4 RNA expression in vitro
Source: RNA. 2019 Sep;25(9):1211–7. doi: 10.1261/rna.070177.118 (PMC6800509; doi:10.1261/rna.070177.118)

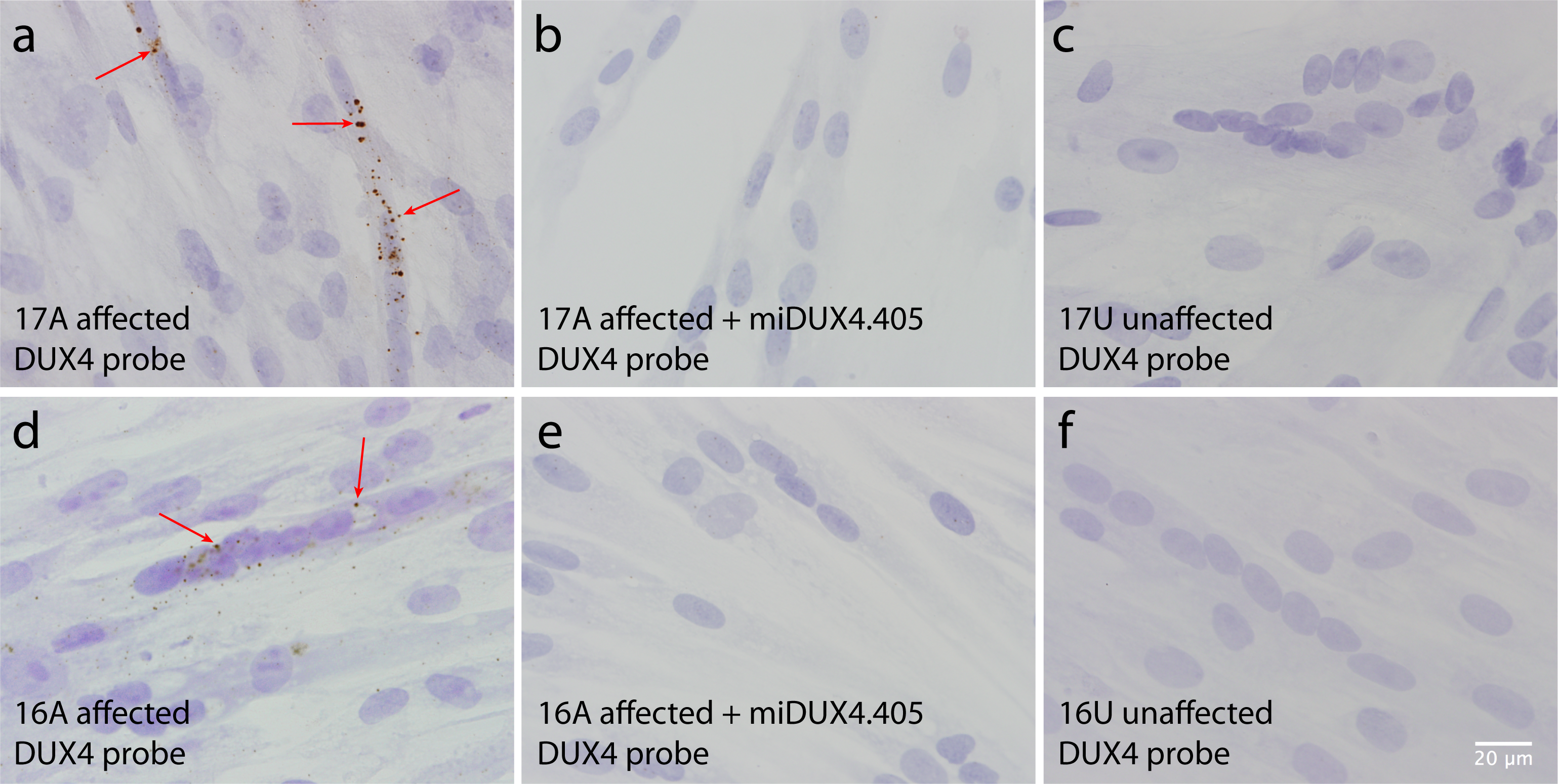

Supplement: Supplemental Material [file supp_070177.118_Supplemental_Figure_1.tif]

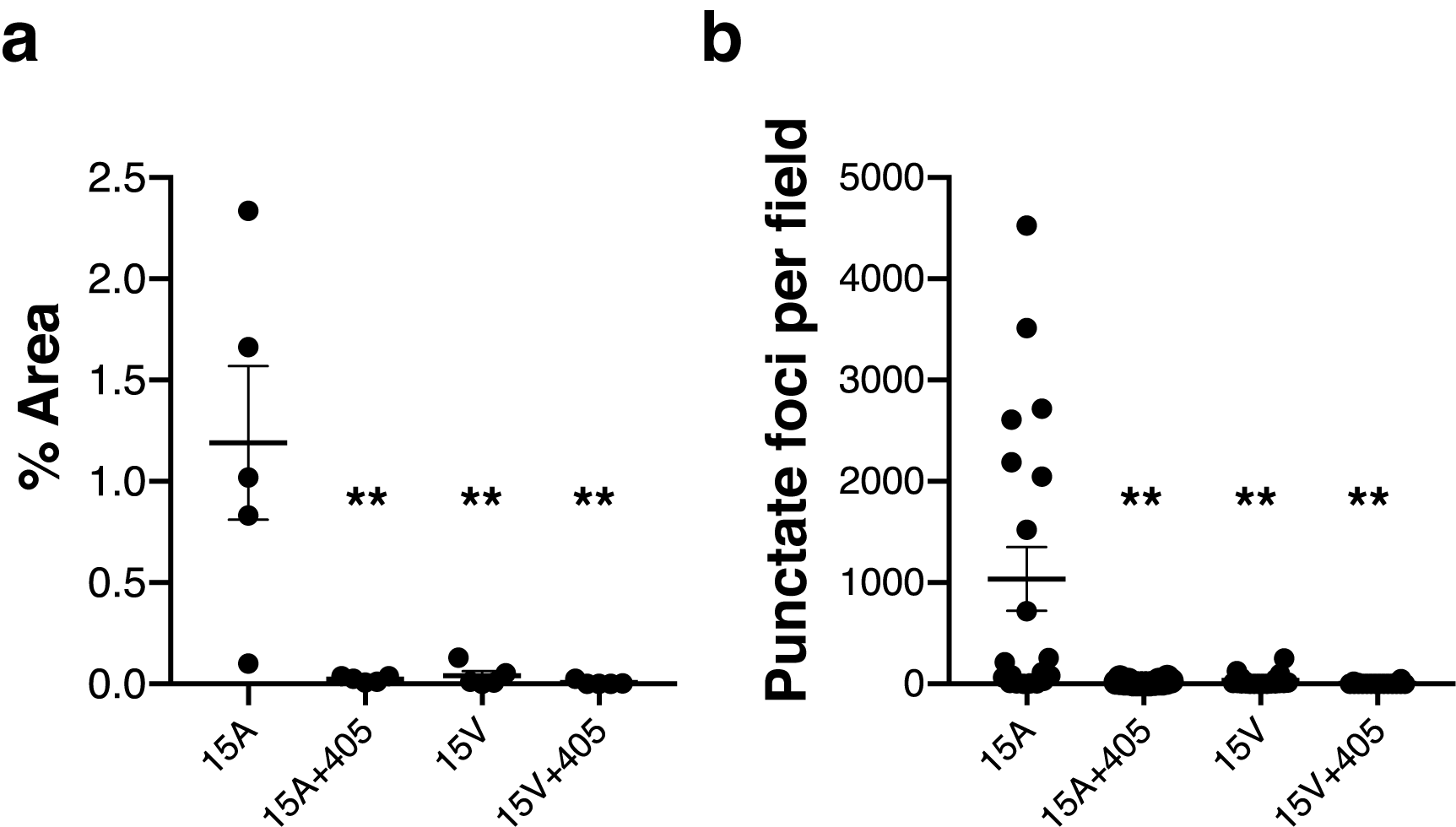

Supplement: Supplemental Material [file supp_070177.118_Supplemental_Figure_3.tif]

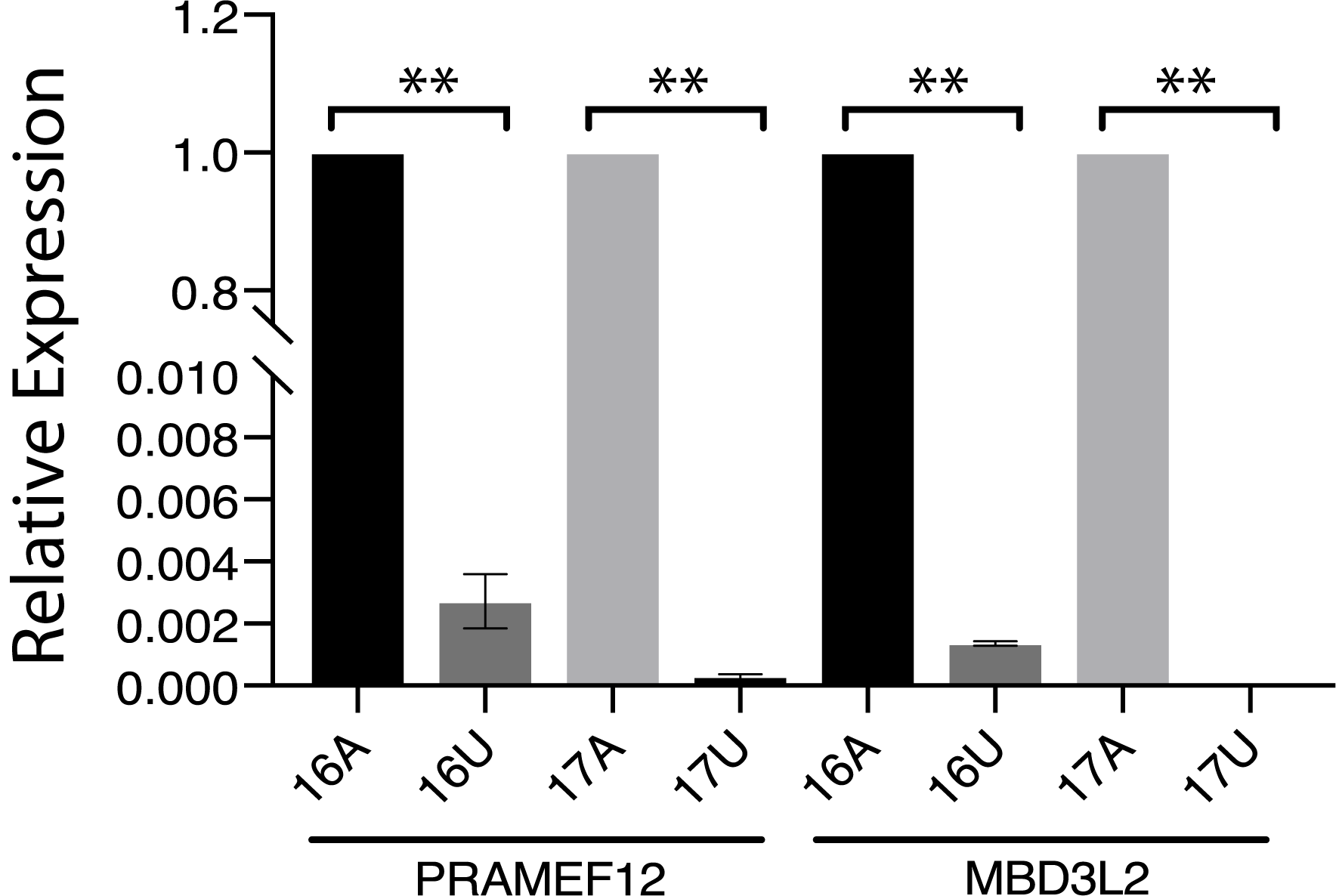

Supplement: Supplemental Material [file supp_070177.118_Supplemental_Figure_4.tif]
