# Supplementary material for: RNAscope in situ hybridization-based method for detecting DUX4 RNA expression in vitro
Source: RNA. 2019 Sep;25(9):1211–7. doi: 10.1261/rna.070177.118 (PMC6800509; doi:10.1261/rna.070177.118)
Supplement: Supplemental Material [file supp_070177.118_Supplemental_Legends.docx]

**Supplemental Figures**

**Supplemental Figure 1. RNAscope detects endogenous DUX4 mRNA in 16A and 17A FSHD myotubes.** FSHD 16A and 17A myotubes demonstrated higher amounts of *DUX4* mRNA compared to non-FSHD 16U and 17U myotubes, as determined by RNAscope staining. All cells shown were stained with *DUX4* RNAscope probe. Arrows indicate brown punctate signal. (a) FSHD affected 17A cells; (b) 17A transfected with mi405; (c) unaffected 17U cells; (d) FSHD affected16A cells; (e) 16A cells transfected with U6.mi405. (f) unaffected 16U cells. Objective 100X. Scale bar, 20 microns.

**Supplemental Figure 2. Video demonstration of signal quantification using Image J.**

DUX4 RNAscope signal (brown stain) was quantified using ImageJ (Fiji) software based on Image J analysis guidelines provided by ACDBio (TS 46-003/Rev A/Date 6212018). The stepwise details below accompany the video in Supplemental Figure 2.

1. Open the original image using Image J software.
2. Activate Weka classifiers by selecting **Plugin > Segmentation > Trainable Weka Segmentation**.
3. Add a new classifier by selecting **Create new class** and provide a name. Here we used “Theme”.
4. Press the **Settings** button on the bottom left of the screen to activate a **Segmentation settings** pop-up menu. in the **Training features** portion, select only the **Mean, Maximum, Variance, Minimum, Entropy.** Under **Class Names** type “Probe” until Class 1, “Nuclei” under Class 2, and “Theme” should already be present in Class 3. Hit the OK button to close the Settings menu.
5. Next use the freehand selection tool (5^th^ button from the left on the selection menu) and select 2-4 color examples for each class (e.g. brown dots for DUX4 signal under the “Probe” class; purple nuclei under the “Nuclei” class; background under “Theme”. Here we used 3 selections for each class. Following selection, press the **Train Classifier** button on the top left of the screen.
6. Wait several seconds or minutes for image processing, then press the **Create result** button near the top left of the screen.
7. Separate the Probe class from other classes by selecting **Image>Adjust>Threshold.** The image here turned red. Then under the **Threshold** pop-up menu, change the threshold range (bottom toggle bar) until the probe class is visible in the image. Select apply and close the window. Here the image turned black and white with only the filtered DUX4 probe data visible.
8. Select the **Analyze Particles** from **Analyze** tab. The default settings should read “0-Infinity” under **Size** and 0.00-1.00 under **Circularity**. Hit OK.
9. The results **Summary** then shows total area of detected signal and also percent of total area.
10. We defined 16 pixels as the minimum visible DUX4-positive punctate focus. Dividing total area of signal by 16 therefore provides an evaluation of DUX4 foci per defined area.
11. Save the classifier and data to a local drive by selecting the **Save classifier** and **Save data** from settings on the Weka classifiers, and use the identical settings for all remaining images.

**Supplemental Figure 3. Independent, blinded confirmation of RNAscope quantification.**

These data represent an independent repeat of the data in Fig 3b and Fig 3c by a second experimenter. Please see Fig 3 legend for details.

**Supplemental Figure 4. DUX4-activated biomarker expression in 16A, 16U, 17A, 17U myotubes.** QPCR shows that mRNAs/cDNAs of DUX4 biomarkers *PRAMEF12* and *MBD3L2* were present in affected 16A and 17A myotubes but absent or extremely low in unaffected 16U and 17U counterparts. ** represents significant differences from untreated 16A or 17A cells; p<0.01, ANOVA.
